# Antibacterial Activities of Bacteria Isolated from the Marine Sponges *Isodictya compressa* and *Higginsia bidentifera* Collected from Algoa Bay, South Africa

**DOI:** 10.3390/md15020047

**Published:** 2017-02-17

**Authors:** Relebohile Matthew Matobole, Leonardo Joaquim van Zyl, Shirley Parker-Nance, Michael T. Davies-Coleman, Marla Trindade

**Affiliations:** 1 Institute for Microbial Biotechnology and Metagenomics (IMBM), Department of Biotechnology, University of the Western Cape, Robert Sobukwe Road, Bellville 7535, Cape Town, South Africa; ry10mat@yahoo.com (R.M.M.); ituffin@uwc.ac.za (M.T.); 2 Department of Zoology, Nelson Mandela Metropolitan University, University Way, Port Elizabeth 6031, South Africa; Shirley.Parker-Nance@nmmu.ac.za; 3 South African Institute for Aquatic Biodiversity (SAIAB), Somerset Street, Grahamstown 6139, South Africa; 4 Department of Chemistry, University of the Western Cape, Robert Sobukwe Road, Bellville 7535, Cape Town, South Africa; mdavies-coleman@uwc.ac.za

**Keywords:** *Isodictya compressa*, *Higginsia bidentifera*, natural products, marine sponge, antimicrobial

## Abstract

Due to the rise in multi-drug resistant pathogens and other diseases, there is renewed interest in marine sponge endosymbionts as a rich source of natural products (NPs). The South African marine environment is rich in marine biota that remains largely unexplored and may represent an important source for the discovery of novel NPs. We first investigated the bacterial diversity associated with five South African marine sponges, whose microbial populations had not previously been investigated, and select the two sponges (*Isodictya compressa* and *Higginsia bidentifera)* with highest species richness to culture bacteria. By employing 33 different growth conditions 415 sponge-associated bacterial isolates were cultured and screened for antibacterial activity. Thirty-five isolates showed antibacterial activity, twelve of which exhibited activity against the multi-drug resistant *Escherichia coli* 1699, implying that some of the bioactive compounds could be novel. Genome sequencing of two of these isolates confirmed that they harbour uncharacterized biosynthetic pathways that may encode novel chemical structures.

## 1. Introduction

The marine environment has been identified as a rich source of bioactive compounds with interesting chemical diversity, and in particular those produced by marine sponges [1]. Sponges belong to the phylum *Porifera*, and are some of the oldest metazoans on Earth [2,3]. There are four classes of marine sponges, namely *Calcarea*, *Hexactinellida*, *Homoscleromorpha* and *Demospongiae* with the *Demospongiae* being most abundant, representing 83% of described species [4]. These filter feeding sessile organisms occur primarily in marine environments where they survive under immense competition and predation [5,6,7].

There is evidence that many of the previously identified marine sponge secondary metabolites are of microbial origin [1,8,9,10]. Their ability to survive in this competitive environment could be due to their own adaptation, in addition to or perhaps because of the microbial communities they harbor, which can account for up to 40% of the sponge’s wet weight [5,7,11]. Furthermore, these microbes are proven to produce medically relevant compounds including ecteinascidin-743 (Yondelis^®^), dolastatin-10 (Adcetris^®^) and halichondrin B (Halaven^®^) [12]. As a result, marine sponge-associated microbes are sought after for natural product discovery [13,14,15,16] due to their potential for delivering novel bioactive compounds [17,18]. In general, the large scale production of microbial-derived secondary metabolites for study and use is hampered by the lack of these bacteria in culture collections, either through an inability to culture them or identify the microbes responsible for producing an observed bioactivity [19]. It is therefore important to develop techniques to cultivate these marine sponge symbionts in order to access and exploit their secondary metabolite capabilities [20]. The use of novel culture media, such as very minimal nutrient content [21], application of diffusion chambers [20,22] or microencapsulation [23], have successfully resulted in the isolation of previously uncultured marine bacteria.

South Africa has a unique coastline that is dominated by two major current systems: the cold Benguela current and the warm Agulhas current [24,25]. As a result of the physico-chemical differences introduced by the two currents, the coastline has an exceptionally rich and varied marine life which remains largely unexplored. The few reported studies performed on South African marine sponges thus far do not take their associated microorganisms into account [26]. The latrunculid sponge, *Tsitsikamma favus*, endemic to the coast of South Africa, represents one of the few specimens whose microbial diversity has been assessed and serves as an example of what South African sponges may have to offer [8]. Unique to this sponge is the production of pyrroloiminoquinones, known as tsitsikammamines, that mediate cytotoxicity through the cleavage of DNA due to inhibition of topoisomerase I [27] and it is thought that these secondary metabolites are of microbial origin [9]. The South African coast therefore represents a potentially resourceful environment for the exploration of novel bioactive compounds.

This study assesses the bacterial diversity associated with five South African marine sponges, namely *Waltherarndtia caliculatum*, *Higginsia bidentifera*, *Isodictya compressa*, *Spongia* and *Axinella* species. All five sponges belong to the class *Demospongiae*. The *Higginsia* genus was first described by Higgin in 1877 during a scientific expedition aboard the ship Argo, with the species *Higginsia bidentifera* described in 1886 and according to the World Porifera Database, appears to be endemic to South African coastal waters [28,29]. *Isodictya compressa* was first described by Esper in 1794, while *Waltherarndtia caliculatum* was described by Kirkpatrick in 1903, both of which are endemic to South Africa [30,31,32,33] and the bacteria associated with these three marine sponges have not been investigated. *Axinella* and *Spongia* species have a global distribution and the microbial populations of some species of these sponge genera have been studied [34,35,36,37]. We first determined the bacterial diversity associated with these five sponges, then isolated bacteria from two of these and demonstrated their ability to produce antibacterial compounds. Here we report a new collection of South African marine sponge-associated bacteria that show antibacterial activity when subjected to a variety of culture conditions.

## 2. Materials and Methods

Unless otherwise specified, chemicals and reagents used in this study were supplied by Sigma Aldrich Chemical Company (Darmstadt, Germany), Merck Chemical and Laboratory Supplies (Darmstadt, Germany) and Kimix Chemical and Laboratory Supplies (Cape Town, South Africa). The enzymes, DNA size markers and polymerases were purchased from Fermentas Life Sciences Ltd. (Vilnius, Lithuania).

### 2.1. Sample Collection

The five marine sponges (*Waltherarndtia caliculatum*, PE05; *Higginsia bidentifera*, PE07; *Isodictya compressa*, PE09; an *Axinella* species, PE11; and a *Spongia* species PE08) were collected in January and April 2013 in Algoa Bay, Port Elizabeth, South Africa (longitude 34°00.366 S and latitude 25°43.209 E; 15 °C), and kept on ice in sterile sea water. The sponge samples (Figure S1) were shipped to the Institute for Microbial Biotechnology and Metagenomics, Cape Town, where the sponge material was stored at –20 °C and –80 °C in 20% glycerol until further processing. Voucher specimens of all sponge samples are housed in the South African Institute for Biodiversity (SAIAB) in Grahamstown. 

### 2.2. Terminal Restriction Fragment Length Polymorphism (T-RFLP) Analysis

The five sponge samples were thawed on an ice/water slurry and cut with a sterile blade into 1 g aliquots for further processing. The UltraClean Soil DNA Isolation kit (MOBIO Laboratories, Carlsbad, CA, USA) was used to isolate DNA according to the manufactures protocol. Metagenomic DNA (mDNA) was further purified as described by Liles et al. [38]. The NucleoSpin^®^ Gel and PCR Clean-up kit (Macherey-Nagel, Düren, Germany) was used to extract the mDNA from the agarose plug.

The PCR reaction mixture to generate amplicons for T-RFLP was as follows: 1 × Phusion DNA polymerase buffer, 0.2 mM dNTP mix, 0.5 µM of each forward primer E9F (5′-GAGTTTGATCCTGGCTCAG-3′) labelled at the 5′ end with fluorescein amidite (FAM) and reverse primer U1510R (5′-GGTTACCTTGTTGTTACACTT-3′), 0.02 U/µL of Phusion DNA polymerase (Fermentas, Lithuania), 100 ng of template mDNA and ultraviolet (UV) treated water [39,40]. The following cycling conditions were used: initial denaturation of 98 °C for 2 min followed by 35 cycles of 98 °C for 20 s, 54 °C for 30 s, 72 °C for 1 min and final elongation at 72 °C for 5 min. Duplicate amplifications were conducted for each sample, pooled and purified using the NucleoSpin^®^ Gel and PCR Clean-up kit (Macherey-Nagel). Amplicons (1 µg) were digested in 30 µL volumes containing 3 µL of 10 × Tango buffer, 2 µL of *Hha*I (10 U/µL), 4 µL *Hae*III (10 U/µL), and UV treated water. Digestions were performed in a Bio-Rad T100 Thermal Cycler (Bio-Rad, Hercules, CA, USA) overnight at 37 °C, followed by inactivation at 80 °C for 20 min. Capillary electrophoresis and analysis of the fluorescently labelled terminal restriction fragments (T-RFs) was carried out on an automated ABI3130XL genetic analyzer (Applied Biosystems, Foster City, CA, USA) by the Central Analytical Facility at the University of Stellenbosch, South Africa. The marker used for the analysis was GS500LIZ. T-RFLP profiles were analysed using freeware Peak ScannerTM Software Version 1.0 (Applied Biosystems). Peak height was used to characterize unique T-RFs. Peaks shorter than 25 bp and larger than 1200 bp were excluded from further analysis. Valid peaks were identified and aligned using the online T-REX software [41] to create an operational taxonomic unit (OTU) data matrix. In theory, each OTU represents an individual T-RF where one T-RF represents one distinct ribotype [42]. It should be noted however that, one peak may represent two different bacterial species or two peaks may be one bacterial species depending on the recognition site of restriction enzymes on the 16S ribosomal RNA (rRNA) gene sequence [43,44,45]. The OTU matrix was analysed using Primer 6, Version 6.1.11 (Primer E, Plymouth, UK) software and diversity indices for all the samples were determined.

### 2.3. Bacterial Culturing

Thawed sponge material (1 g) was homogenised by grinding with a sterile pestle and mortar in 9 mL of sterile sea water. The sponge homogenates were serially diluted in sterile sea water to 10^−6^, and from each dilution, 100 μL aliquots were plated on 23 general media (Table S1). The full culturing scheme is depicted in Figure S2. In addition, aliquots of the sponge homogenates underwent heat treatment to select for endospore forming bacteria [15] and to inhibit the growth of Gram negative fast growing bacteria [46]. The heat treatment process was adapted from Phelan et al. (2012) [15] with minor modifications. An aliquot of 100 μL of the 10^−1^ dilution of the sponge homogenate was incubated at 80 °C for 10 min and serially diluted to 10^−6^. Aliquots of 100 μL were aseptically plated on three media Shivji Nutrient Agar (SNA), Tryptic Soy Agar (TSA) and Zobell Agar (ZBA). Liquid enrichment was used to select for cyanobacteria [47] and nitrogen fixing bacteria [48]. For liquid enrichment cultures, 100 μL of the 10^−1^ dilutions of the sponge homogenate were added to 9 mL of Ashby’s Nitrogen Free Media (ANFA), MagMin Semi-Solid Medium (MMM) and Blue Green Algae (BG11) liquid media. The liquid enrichment cultures were incubated at 15 °C for up to six weeks on a shaker at 150 rpm. Subsequently, 100 μL of the liquid enriched cultures were plated on ZBA plates, as ZBA is one of the rich media which can generally support proliferation of a range of different bacteria. After the initial isolations, three media Glucose Yeast Media (GYM), TSA and ZBA were selected as the “standard/rich media” for routine culturing and characterization of the bacterial isolates. Isolates from Trypticase Soya Agar pH 4.5 (TS4), TSA and SNA, inclusive of those which underwent different treatments, were cultured on TSA. Isolates from Medium A Agar (MAA), GYM, Modified 172F medium (172), Oatmeal Agar (OMA), Actinomyces Isolation Medium (AIM), and Planctomycetes Medium (PMM) along with their different treatments were cultured on GYM. Isolates from the thirteen other media with their respective treatments were also cultured on ZBA. Antibiotic treated sponge homogenates were prepared by adding 20 μg/mL of streptomycin [49] to the homogenate. The homogenates were agitated on a shaker (150 rpm) for 1 h at room temperature. The sponge homogenates were centrifuged at 11,000× *g* for 3 min and the supernatant was discarded. Sterile sea water (1 mL) was used to re-suspend the pellet and the suspension was serially diluted to 10^−6^. Aliquots of 100 μL of the 10^−1^ to 10^−6^ dilutions were plated on eight media (OMA, OMA + NaCl (1.8% wt/vol), AIM, GYM, 172, MAA, Yeast Extract-Malt Extract (YEME) and Starch Casein Nitrate Agar (SCN)) containing cyclohexamide (100 μg/mL) and nalidixic acid (50 μg/mL) [50]. All plates were incubated at 15 °C for up to four weeks. Colonies were examined and picked on a daily basis selecting for colonies with different morphologies (shape, colour, texture, and size).

### 2.4. Antimicrobial Screening

The marine sponge-associated isolates were screened, using agar overlay assays, for antibacterial activity against the Gram-positive strains *Mycobacterium smegmatis* LR222, *Bacillus cereus* ATCC10702, *Staphylococcus epidermidis* ATCC14990 and the Gram negative strains *Pseudomonas putida* ATCC27853 and the multidrug resistant *Escherichia coli* 1699 (Cubist, Lexington, MA, USA). Isolates were cultivated on four media (ZBA, GYM, TSA and Activated Charcoal Medium (ACM)) for two weeks at room temperature. On the day prior to carrying out the overlay assays, the test strains were cultured overnight in 10 mL Luria Broth (LB) at 37 °C with aeration at 250 rpm. To ensure that approximately the same concentration of cells (using optical density measurements, OD_600_) were present in the sloppy agar, the following formulae were used to calculate the volume of culture to add to 6 mL of sloppy agar (LB): OD_600_ × X μL = 4 (for *E. coli*). OD_600_ × X μL =160 (for other test strains). The sloppy agar containing the test strains was poured over the marine isolate growth, incubated at 30 °C and examined for zones of inhibition after 16 h and again after 48 h.

For induction of antimicrobial activities, a one-strain-many-active-compounds (OSMAC) approach was followed. A total of 29 isolates were selected; 12 that gave hits against the panel of test strains in the overlay assays and an additional 17 isolates that did not show antibacterial activity in the first round of screening (Table S2). A matrix incorporating the following growth modifications or treatments was developed: heat or cold shock treatment, co-culturing with sponge-associated bacteria, and varying carbon (mannitol, succinic acid and starch at 5 mM) and nitrogen (NH_4_Cl or NaNO_3_ at 0.02% *w*/*v*) sources or phosphate concentrations (KH_2_PO_4_ at 0.1 µM or 0.5 mM). The base medium employed was a minimal media containing per liter: 18 g NaCl, 2 g MgCl_2_, 0.525 g KCl, 0.075 g CaCl and 2.38 g HEPES buffer. The experiment was set up in a 96-well format on large petri dish plates (20 mm × 150 mm). The method of plating in 96-well format was similar to that described in [51] with some modification. Single colonies from the selected isolates were inoculated into 96-well plates containing 70 µL sterile water in each well, and shaken at 100 rpm on a shaker for 10 min at room temperature (RT). Cultures were then plated aseptically on the different matrix media using a 96-pin hedgehog and allowed to grow for 2 weeks at RT. The order of inoculation of isolates was interchanged between isolates that gave positive hits on standard medium in the first round of screening and those which did not. All isolates were inoculated in triplicate on each 96-well plate and therefore located at different positions resulting in different neighboring isolates which resulted in a co-culture setup (Figure S3). On the day prior to performing overlays, cold and heat shock treatments were carried out. The heat shock method used was adapted from [52] with minor changes. Heat shock plates were incubated for 1 h at 42 °C, whereas cold shock treatment was carried out at 4 °C for 1 h. The heat and cold shock treatments were repeated on the day of the antibiotic overlay assay. The day prior to carrying out the overlay assay, single colonies of each of the test strains was inoculated into Luria Broth (10 mL) and the cultures were incubated at 37 °C overnight while shaking at 250 rpm. The optical density (OD_600_) of the cultures were determined after which they were diluted to achieve the same overall concentration of cells. An overlay assay similar to that described above was carried out using 24 mL of agar for the large format plates (20 mm × 150 mm).

### 2.5. DNA Sequencing and Phylogenetic Analysis

For 16S rRNA identification of the bioactive isolates, genomic DNA extraction was performed using the GeneJET Genomic DNA Purification Kit (Thermo Scientific, Waltham, MA, USA). A single colony was inoculated in 10 mL TSA broth or ¼ strength ZBA broth and incubated on a shaker (150 rpm) at room temperature for 2 to 5 days depending on growth rate. DNA extraction was carried out according to the manufacturer’s instructions for Gram-negative bacteria. Minor modifications were made for Gram-positive bacteria where 200 µL of lysis buffer (25 mM Tris-HCl pH8, 50 mM glucose, 10 mM EDTA and 25 mg/mL lysozyme) was added to the pelleted bacterial cells and the solutions were incubated at 37 °C for 30 min. Thereafter the manufacturer’s instructions were followed. PCR amplification was performed in 50 µL volumes which consisted of 1 × Dream Taq buffer, 0.2 mM of dNTP mix, 1 µM of each of the reverse and forward primers, 100 ng template DNA, 1.25 U DreamTaq DNA polymerase (Fermentas), and sterile distilled water. The universal primers E9F and U1510R were used to amplify the 16S rRNA gene sequences of the marine sponge-associated bacteria. The following cycling conditions were used: initial denaturation 95 °C for 5 min, 35 cycles of 95 °C for 45 s, 55 °C for 45 s, 72 °C for one and a half min and final elongation of 72 °C for 10 min.

The DNA sequence determination of purified 16S rRNA gene products were carried out by the Central Analytical Facility at Stellenbosch University using an ABI PRISM 377 automated sequencer (Applied Biosystems). The data from the sequencing unit was processed using Chromas Pro 1.5a software (Technelysium, South Brisbane, QLD, Australia) for alignment and manual editing of sequences. The neighbor-joining phylogenetic tree was constructed using MEGA 7 software [53]. Bootstrap tests were performed 1000 times.

### 2.6. Genome Sequencing

Whole genome sequencing of PE09-72 and PE09-221 was performed at the sequencing facility within the Institute for Microbial Biotechnology and Metagenomics, University of the Western Cape, South Africa. Genomic DNA was prepared using standard phenol:chloroform extraction. Sequencing libraries were prepared using the Illumina Nextera XT library prep kit according to the manufacturer’s instructions and sequencing was performed using the Illumina MiSeq Reagent kit V3 (Illumina, San Diego, CA, Country) which included a 10% phiX V3 spike [54]. This resulted in 2 × 300 base pair (bp) paired end reads (forward and reverse). Analysis of the sequence data was carried out using CLC Genomics version 7.5.1 (CLC, Aarhus, Denmark). Raw reads were reference assembled to phiX V3 genome used in the sequencing reaction for quality control. Unmapped reads were mapped to the human genome to remove any contaminating sequences. A de novo assembly was carried out on the remaining unmapped reads using default parameters. Basic analysis of the assembled contigs were carried out using the RAST server [55] and BLAST analysis against the NCBI database [56]. The genomes have been deposited in the Genbank database under accession numbers MIJA00000000 and MIJB00000000 for PE09-72 and PE09-221 respectively.

## 3. Results and Discussion

### 3.1. Bacterial Diversity Analysis and Culturing of Marine Sponge-Associated Bacteria 

**Figure 1:**
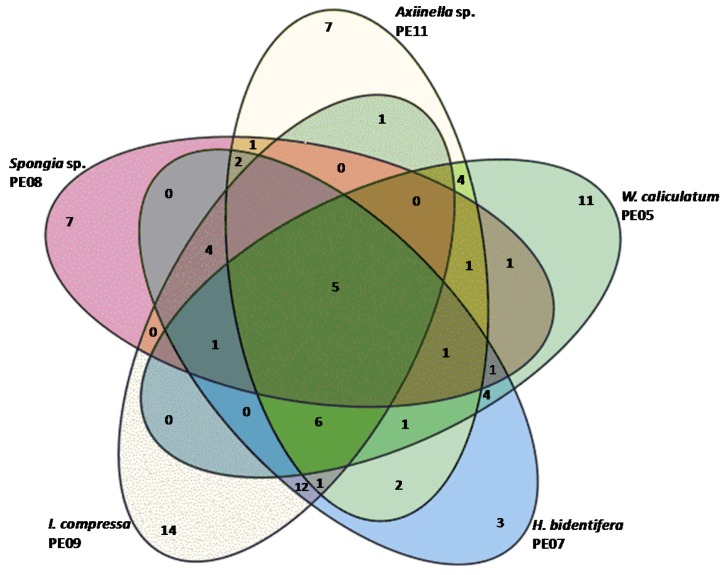
Venn diagram of species specific and shared operational taxonomic units (OTUs) (at genus level) in the selected marine sponges.

Five sponges (PE05, PE07, PE08, PE09, PE11) were investigated for their bacterial diversity, using 16S rRNA based T-RFLP analysis to identify those with highest diversity and unique operational taxonomic units (OTUs) to improve the chance of isolating novel bacteria. T-RFLP profiles revealed that there was a total of 179 OTUs from the five marine sponges, 90 of which were unique (Figure 1). Studies have classified microbial OTUs as either species specific OTUs (defined as OTUs unique to a certain sponge species) or sponge specific OTUs (defined as OTUs found in sponges but not found in other environments) [57,58]. Notably, five of the OTUs were shared among all five sponges indicating that the representative OTUs could be sponge specific [59,60].

The Simpson index of the bacterial species from PE05 and the PE11 was the least even, indicating the presence of more dominating OTUs in these samples, while the other sponges had a more even distribution of species abundance (Table 1). PE07 and PE09 yielded the most OTUs with 43 and 44 respectively and had the highest evenness. Although seemingly low, the OTUs from the present study were similar to the numbers of T-RFLP-based OTUs reported from other studies where 45 and 32 OTUs were identified in *Hymeniacidon heliophila* and 36 and 22 OTUs were identified in *Haliclona tubifera* depending on the restriction enzymes used [61]. PE09 had the highest number of species specific OTUs (14) with the second highest being PE05 with 11 OTUs. Although PE07 had the second highest number of OTUs only three were specific to this sample which indicated that fewer *H. bidentifera-*specific OTUs were isolated from the sample. Given the higher diversity and even representation of species present in both PE07 and PE09, as well as the uniqueness of PE09 profiles and the representative nature of PE07, these were selected for bacterial isolation studies.

**Figure 2:**
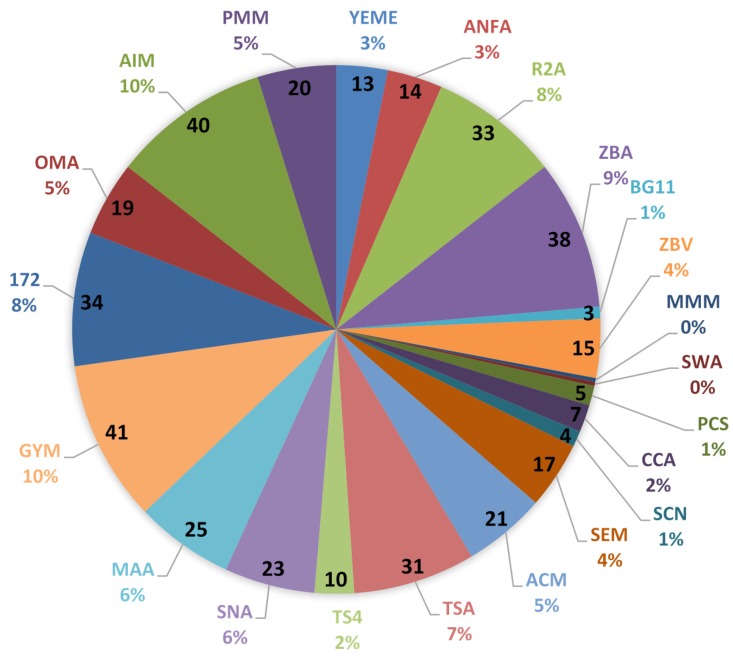
Total number (and percentage) of bacterial isolates cultured from the marine sponge samples PE07 and PE09 per medium type. The number shown inside each pie section indicates the raw number of isolates from that medium type. Only 1 isolate was recorded from MMM and SWA respectively.

A total of 415 unique bacterial colonies, as judged by their morphology (size, shape, colour and texture), were isolated from the two sponge samples with PE09 yielding 273 isolates and 142 bacterial isolates from PE07, respectively. The fewest number of isolates were obtained from samples growing in liquid enrichment medium and from the heat treated medium, which yielded 5 and 12 isolates respectively. This could be due to the selectivity in the liquid enrichment medium which was meant to select for nitrogen fixing bacteria. In the case of heat treatment, the low viable microbial numbers post treatment indicate that the number of spore-forming bacteria are low in these marine sponges or that sporulation had not been induced in spore formers at the time of harvest [15]. The antibiotic treatment yielded 37 isolates while the non-treated medium resulted in 361 bacterial isolates. GYM was the medium which resulted in the highest number of bacterial isolates (40) which accounted for 10% of the total isolates. AIM and ZBA resulted in 39 (10%) and 37 (9%) isolates respectively (Figure 2). The medium which resulted in the fewest bacterial isolates were MMM and Seawater Agar (SWA) since each contributed only one bacterial isolate. Both media discourage growth of fast growing bacteria thus resulting in fewer numbers of microbial isolates.

### 3.2. Antimicrobial Screening and Induction

The antibacterial properties of microbial isolates from PE07 and PE09 were investigated in an agar overlay assay against five test strains (Table 2). Most isolates only showed a narrow spectrum of activity, being active against only one test strain when cultured on standard media (ZBA, GYM, TSA and ACM). However, isolates PE07-7, PE07-144, PE07-207, PE09-87, PE09-105 and PE09-119 displayed activity against two or more test strains. Notably, PE07-7 and PE09-119 only displayed activity against Gram-positive test strains, while PE07-144, PE07-207 and PE09-105 had activity against both Gram-positive and Gram-negative test strains. Most activity was recorded against *B. cereus* with 17 out of 32 strains showing activity against this test strain, while seven isolates gave activity against the highly antibiotic resistant *E. coli* 1699 (resistant to 52 known antibiotics). It was also noted that none of the isolates displayed antibacterial activity on more than two growth media, suggesting inducible genes or pathways. It is also important to note that all isolates grew to some degree on the media used and lack of activity is not due to the inability of the isolate(s) to grow.

To further induce antimicrobial activities, an OSMAC approach was taken for a subset of the isolates. From screening 29 microbial isolates in the matrix of 36 culture/treatment conditions and co-culture, six isolates showed induced antibacterial activities (Table 3). Most notably, isolate PE09-72 produced activity against all test strains in mannitol containing medium, especially with the addition of heat shock, whereas only one activity was produced with succinic acid as the carbon source. This indicates that the pathway(s) responsible for the activities are likely subject to carbon catabolite repression (CCR) [62]. Although no medium composition induced activities against all test strains, some medium compositions (NH_4_Cl-LP, NH_4_Cl-HP and NaNO_3_-HP) induced activity in PE09-72 against four of the test strains when mannitol was used as carbon source and the culture heat shocked.

Differential antimicrobial activities were observed in relation to the media the isolates were cultured on. For instance, the isolate PE07-5 showed antibacterial activities against *E. coli* 1699 and *M. smegmatis* in GYM medium and only against *B. cereus* on ZBA medium. This is a well-known phenomenon regarding microbial secondary metabolite production [8,63].

### 3.3. 16S rRNA Gene Sequence Analysis of Bacteria Producing Antimicrobial Activity

Twenty-six of the bacterial isolates which showed antibacterial activities against the test strains were identified by determination of the sequence of the 16S rRNA gene sequence (Table 4). The identities ranged from 96.5% to 100% identity over a region of 1340 bp and the bacteria belonged to the following phyla: *Actinobacteria*, *Firmicutes* and *Proteobacteria* (*Alphaproteobacteria* and *Gammaproteobacteria*). Unexpectedly the *Firmicutes* and *Gammaproteobacteria* were the phyla which showed the most antibacterial activities as opposed to the *Actinobacteria*, well-known for their antibiotic activities [9,64,65].

In some instances, isolates that shared 99% 16S rRNA gene sequence identity differed in their bioactivity profiles and were thus considered to be different OTUs. An example of this was observed with isolates PE09-100 and PE09-110 which were both identified as *Citricoccus nitrophenolicus* whereas their bioactivities differed (Table 2). Similar analyses of five strains of *Pseudoalteromonas luteoviolacea* revealed that the strains were closely related based on 16S rRNA gene sequences however they produced different bioactivity profiles [66,67].

Isolate PE09-72, which showed most activity as described above, was identified as a *Bacillus zhangzhouensis* strain, a newly described bacterial species [68] that belongs to the *Bacillus pumilus* clade, a bacterium known for its antibiotic producing ability [69,70,71,72]. *Bacillus marisflavi*, *Psychrobacter alimentarius* and *Arthrobacter citreus* which PE07-7, PE09-87 and PE09-119 were identified as respectively, are here, for the first time, shown to display antimicrobial activity. *Arthrobacter citreus* is often used as test organism against which to test for antimicrobial activity, yet here it displays antibacterial properties against both Gram-positive and Gram-negative bacteria [73,74], while *B. marisflavi* and *P. alimentarius* are relatively newly described species [75,76].

**Figure 3:**
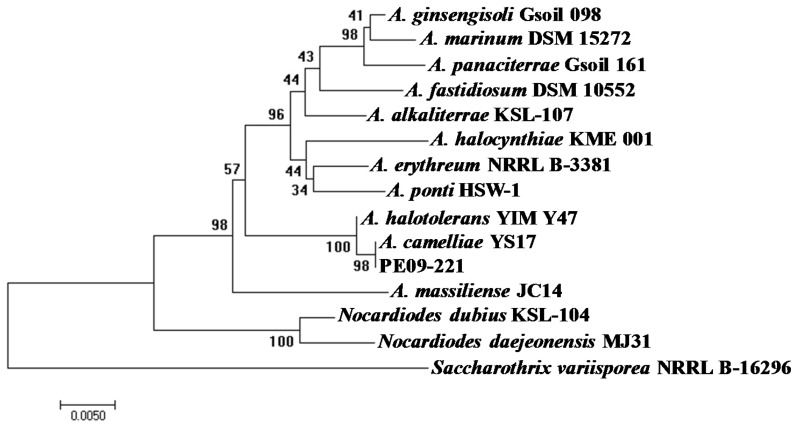
Neighbor-joining tree generated by analysing partial (approximately 1340 bp) 16S rRNA gene sequences of *Aeromicrobium sp*. compared with isolate PE09-221.

The most novel isolate at the time of isolation was PE09-221, which has 96.5% identity to *Aeromicrobium erythreum.* It has subsequently been shown to be a strain of the recently described *Aeromicrobium camelliae* species which is also closely related to another newly described species *Aeromicrobium halotolerans* (Figure 3) [77,78]. Thus, our study represents only the second isolation of this species. Of the thirteen described *Aeromicrobium* species, four have been isolated from marine environments with one, *Aeromicrobium halocynthiae,* isolated from a marine ascidian. *A. camelliae*, to which PE09-221 is most closely related, was isolated from Pu’er tea demonstrating that these organisms are not sponge specific and can occupy a wide range of habitats.

### 3.4. PE09-72 and PE09-221 Genome Sequence and Secondary Metabolism

Two isolates were selected for whole genome sequencing: PE09-72, which was the most bioactive isolate under the widest range of test conditions; and PE09-221, due to its novelty at the 16S rRNA sequence level and no genome sequence being available for any isolate of this species. 

The draft genome sequence of PE09-72 is 3.69 Mbp consisting of 109 contigs with an N50 value of 68,385 bp and the largest contig was 316,332 bp. BAGEL3 and antiSMASH analysis of the contigs revealed the presence of eight putative secondary metabolite pathways which could be responsible for the range of activities observed. These include three nonribosomal peptides (NRP), one of which is a hybrid nonribosomal peptide/polyketide operon; a terpene; two bacteriocins; a Type III polyketide and a predicted siderophore-terpene hybrid. Derivatives of all these pathways can be found in *Bacillus* genome sequences currently on the Genbank database with similarity between 40% and 85%. The hybrid NRP/PKS pathway shows weak similarity with the Zwittermycin-A pathway and likely produces a novel compound.

**Figure 4:**
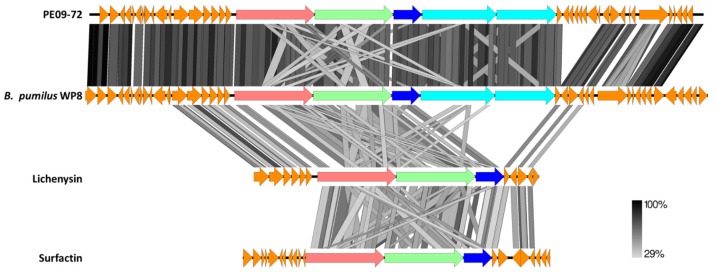
**tBLASTx** comparison of lichenysin-like pathway identified in PE09-72 with the lichenysin and surfactin pathways as well as the most similar pathway identified on a genome currently on Genbank. Gene annotations are as follow: Pink—condensation domain containing protein; Green—condensation domain containing protein; Dark blue—condensation domain containing protein which includes a thioesterase domain; Light blue—two “extra” genes present in the pathway from PE09-72 which contain ketoreductase domains, the downstream gene also contains a thioesterase domain; Orange—other ORF’s (biosynthetic and transport related) identified by antiSMASH which delineates the extent of the pathways. Similarity at the amino acid level in the repeated domain structures results in the criss-cross pattern observed when comparing the biosynthetic genes.

Two of the pathways are related to known antimicrobial compound producing pathways, namely lichenysin and bacilysin, showing 85% similarity in each case. Interestingly the lichenysin-like pathway contains two biosynthetic genes in addition to those found in the characterized lichenysin and related surfactin pathways (Figure 4). Each of these genes harbours a ketoreductase (KR) domain, which is an unusual part of the domain structure of NRP biosynthetic genes. However, when present, they are thought to perform chiral reduction of α-ketoacyl-S carrier proteins generating an ester bond [79,80]. Taken together, the additional biosynthetic genes and unusual domain structure indicate that a novel lichenysin-like compound could be produced from this pathway. These same two open reading frames can be identified in similar pathways on several *Bacillus* species genomes currently on the Genbank database, indicating that this modified, uncharacterized pathway is likely common in a range of *Bacilli*. Even so, differences can still be observed between the PE09-72 pathway and its closest relative (Figure 4). 

Of the seven genes necessary (*bacA*-*E*, *ywfA* and *ywfG*) for production of bacilysin, only six appear to be present. A *bacE* homologue (bacilysin export protein) could not be detected on the PE09-72 genome. Considering the genes downstream (short chain dehydrogenase, NAD-dependent epimerase, glucose-1-phosphate adenylyl/thymidylyl transferase, glycosyltransferase, aldehyde dehydrogenase and polyprenol-monophosphomannose synthase) of the bacilysin-like genes, it is possible that they could form part of a larger pathway than that delineated by antiSMASH to produce a bacilysin-like compound.

As mentioned previously, the pathway(s) responsible for the observed antibacterial activity in PE09-72 may be subject to CCR. A search for the *B. pumilus cre* (WTGNAARCGNWWWCA) consensus [81] indicated a potential site (TTGAAAACGAATTCA) located between two ORFs (14,389–14,403 bp) on contig 33 on which the bacilysin-like pathway was identified with a beta-glucoside transporter located 7 kb downstream on the same contig. Bacilysin production in *Bacillus subtilis* is however not subject to glucose repression [82]. Another putative *cre* site (ATGTAAGCGGTAACA) was identified directly upstream (5936–5950 bp) of a four ORF operon, with the translational start at 5968 bp, predicted to be part of the bacteriocin cluster on contig 38. The ORFs encode an acetyl-CoA acetyltransferase, a 3-hydroxybutyryl-CoA dehydrogenase and two acyl-CoA dehydrogenases. No *Bacillus*-like CodY binding sites (AATTTTCWGAAAATT) could be identified in any of the secondary metabolite pathways identified, nor could the MtlR (Transcriptional activator of mannitol metabolism) recognition site (TTGTCACANNNNNTGTGCCAA) be identified anywhere except upstream of the *mtlR* gene. The observation that activity was turned on against all test strains under mannitol, as opposed to little or no activity on succinic acid, either suggests that one pathway, the product of which shows broad-spectrum activity, was activated, or demonstrates that several of the pathways are subject to CCR.

BAGEL3 identified the presence of a plantazolicin-like peptide and the pathway responsible for its maturation (contig 17 from 98,257 bp to 108,183 bp). These small ribosomally encoded peptides belong to the thiazole/oxazole-modified microcins (TOMM) class of natural products and show a very narrow-spectrum of activity against related *Bacillus* species. Based on the similarity in the core region of the precursor peptide as well as high amino acid identity with the enzymes involved in maturation of the peptide from other *B. pumilus* strains, PE09-72 likely produces the exact same molecule described by Molohon and coworkers [83]. It is therefore probable that the activity against *B. cereus* observed here could be as a consequence of the production of this molecule.

The draft genome of PE09-221 (*A. camelliae*) is 4.01 Mbp and consists of 128 contigs greater than 450 bp with an N50 value of 147,047 bp, with the largest contig being 284,975 bp. The genome sequence of PE09-221 represents the first genome for an *A*. *camelliae* isolate. The G+C content is 67.5% in line with that found for *A. camelliae* (66%), but lower than for most other *Aeromicrobium* species 71%–74% [84,85], with the exception of *A. halotolerans* (44.7%)*.* As intra genus G+C content does not vary greatly (±10%), the low G+C content observed in *A. halotolerans*, suggests that the genus may yet be split into more genera [80,86]. As no genome sequence for *A*. *camelliae* or *A. halotolerans* are available for comparison, comparison of the PE09-221 genome could only be made with the next closest relatives as identified through comparison of the 16S rRNA sequence. Average amino acid identity is 64.8% when compared with *A. erythreum* AR18 and 64.4% when compared with *Aeromicrobium massiliense* JC14. Similarly, the average nucleotide identities were well below the 94-96% cut-off used to delineate new species with values of 79.1% and 78.7% when compared with *A. erythreum* AR18 and *A. massiliense* JC14, respectively [87]. This shows the utility of culturing, despite its limitations, in still isolating novel species. Unlike many other actinobacteria, *Aeromicrobium* species are not well known for their antibiotic production, with the exception of the broad-spectrum macrolide, erythromycin (*A. erythreum)*. PE09-221 did show activity against *B. cereus* and it is possible that one of the two NRP pathways present, one of which is a NRP/PKS hybrid, are responsible for this observed activity. Neither pathway shows similarity to any currently characterized pathway.

## 4. Conclusions

Here we report the first study of bacteria associated with two endemic South African marine sponges and provide the first draft genome for the recently described species *A. camelliae*. Although no entirely novel bacteria were isolated, many of those that were are related to recently described and therefore mostly uncharacterized species. This suggests that these sponges harbour species not routinely isolated. As evidenced by the antimicrobial activities observed for the bacterial isolates and the genome sequences of two of these isolates, endemic South African marine sponges could offer a wealth of new, microbial derived chemistry. In particular, the range and number of the pathways identified for PE09-72 indicates that it is an organism with an impressive arsenal, subject to stringent control. The study serves to further demonstrate the utility of culture-dependent approaches as a valuable tool to tap into marine-derived natural products.

## Figures and Tables

**Table 1:** Diversity indices of the sponge-associated microbial populations.

Sample ID	Marine Sponge ID	Species Specific OTUs	S	1 − λ
PE05	*W. caliculatum*	11	36	0.6923
PE07	*H. bidentifera*	3	43	0.9388
PE08	*Spongia (spongia)* sp. 001RSASPN	7	24	0.8514
PE09	*I. compressa*	14	44	0.9306
PE11	*Axiinella* sp. 007RSASPN	7	32	0.7706

*Index*: species richness (S) = OTUs; Simpson index (1 − λ) = evenness.

**Table 2:** Antibacterial activities against test strains in standard medium.

Isolate	Test Strains				
	Gram Negative		Gram Positive		
	*E. coli* 1699	*P. putida*	*M. smegmatis*	*B. cereus*	*S. epidermidis*
PE07-5	GYM		GYM	ZBA	
PE07-7				TSA, ZBA	TSA, ZBA
PE07-86				TSA	
PE07-105	GYM				
PE07-133	GYM				
PE07-143				TSA, ZBA	
PE07-144	GYM		GYM		
PE07-172					TSA
PE07-200		GYM			
PE07-201		GYM			
PE07-204		GYM			
PE07-207	GYM			GYM	
PE09-7					TSA
PE09-73		ACM			
PE09-87		GYM	GYM		GYM
PE09-100				ZBA	
PE09-105	GYM, TSA		TSA, GYM		TSA
PE09-108				GYM	
PE09-110	ZBA				
PE09-119			ZBA, GYM	TSA, ZBA	ACM, TSA
PE09-124			ACM		
PE09-140			ACM		
PE09-142				ACM	
PE09-168				ACM	
PE09-197				ACM	
PE09-210				ACM	
PE09-213				ACM	
PE09-221				ZBA	
PE09-228				ACM	
PE09-229			ACM		
PE09-235				ACM	
PE09-266				ZBA	
Total hits	7	5	8	17	6

**Table 3:** Antibacterial activities of six selected sponge-associated isolates under matrix screening conditions. Three of the isolates did not display activity in the initial screening, whereas the other three isolates did. RT: Room Temperature (27 °C); HS: Heat Shock treatment (42 °C); CS: Cold shock treatment (4 °C).

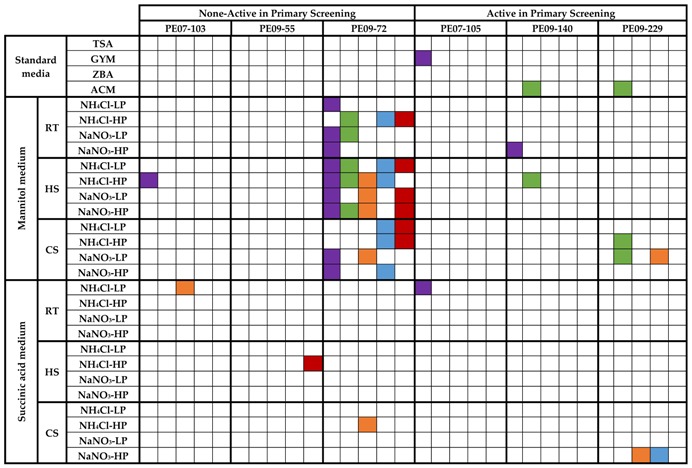
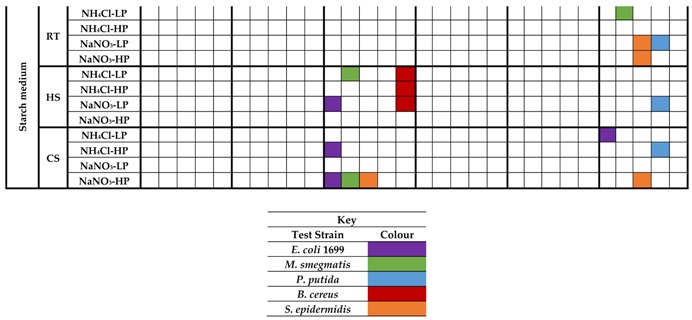

**Table 4:** 16S rRNA gene taxonomic affiliation of 26 of the sponge-associated bacterial isolates showing antibacterial activities against one or more indicator strains.

Phylum/Class	Closest Relative (as Present in EzTaxon)	Identity (%)	Isolate	Isolation Medium
*Actinobacteria*	*Aeromicrobium erythreum* NRRL B-3381	96.5	PE09-221	GYM
	*Arthrobacter citreus* DSM 20133	99.9	PE09-119	ACM
	*Brevibacterium luteolum* CF87	100	PE09-168	GYM
	*Citricoccus nitrophenolicus* PNP1	99.7	PE09-100	SCN
	*Citricoccus nitrophenolicus* PNP1	99.8	PE09-110	172
	*Curtobacterium oceanosedimentum* ATCC 31317	99.3	PE09-7	GYM
	*Micrococcus yunnanensis* YIM 65004	99.7	PE07-133	R2A
*Firmicutes*	*Bacillus kochi*i WCC 4582	99.5	PE07-103	MAA
	*Bacillus marisflavi* TF-11	100	PE07-7	ZBA
	*Bacillus vietnamesis* B-23890	99.7	PE07-5	ZBA
	*Bacillus zhangzhouensis* DW5-4	99.9	PE09-72	ZBA
	*Bacillus vietnamesis* B-23890	99.7	PE07-144	ZBA
	*Oceanobacillus picturae* LMG 19492	99.5	PE07-105	MAA
	*Oceanobacillus picturae* LMG 19492	100	PE09-108	ZBA
	*Sporosarcina aquimarina* SW28	98.8	PE07-172	172
	*Staphylococcus cohnii* ATCC 29974	100	PE07-204	ZBA
	*Staphylococcus epidermidis* ATCA 1490	99.9	PE07-201	TSA
	*Staphylococcus warneri* ATCC 27836	99.9	PE07-200	TSA
*Gammaproteobacteria*	*Halomonas titanicae*	99.9	PE09-210	CCA
	*Halomonas titanicae*	99.9	PE09-228	SEM
	*Halomonas titanicae*	99.9	PE09-229	SEM
	*Kushneria pakistanensis* NCCP-934	98.9	PE09-73	PMM
	*Kushneria pakistanensis* NCCP-934	98.9	PE09-124	ACM
	*Kushneria pakistanensis* NCCP-934	98.9	PE09-266	ANFA
	*Pseudomonas fulva* NBRC 16637	100	PE09-140	SNA
	*Pseudomonas fulva* NBRC 16637	100	PE09-197	ANFA
	*Psychrobacter alimentarius* JG-100	99.9	PE09-87	ACM
*Alphaproteobacteria*	*Pseudovibrio ascidiaceicola* DSM 16392	100	PE09-55	TSA

## Supplementary Materials

The following are available online at www.mdpi.com/1660-3397/15/2/47/s1, Figure S1: Sponges used in this study: PE05—*Waltherarndtia caliculatum*, PE07—*Higginsia bidentifera*, PE08—Spongia sp., PE09—*Isodictya compressa*, PE11—*Axinella* sp., Figure S2: Flow diagram showing the culturing process used in this study, Figure S3: 96-well plate setup for matrix screening. Bioactivities of the isolate PE09-72 when cultured on Na5HM under co-culture conditions with 3 and 4 “immediate” neighbouring isolates in 96 well-plate set up, Table S1: Media used to isolate sponge symbionts, Table S2: Panel of 29 isolates selected for the matrix study; 12 isolates which showed antibacterial activities after primary screening and 17 isolates which did not show any bioactivities.
