# Supplementary material for: Antibacterial Activities of Bacteria Isolated from the Marine Sponges Isodictya compressa and Higginsia bidentifera Collected from Algoa Bay, South Africa
Source: Mar Drugs. 2017 Feb 17;15(2):47. doi: 10.3390/md15020047 (PMC5334627; doi:10.3390/md15020047)
Supplement: Supplementary file 1 [file marinedrugs-15-00047-s001.pdf]

Antibacterial Activities of Bacteria Isolated from the Marine Sponges *Isodictya compressa* and *Higginsia bidentifera* Collected from Algoa Bay, South Africa

Relebohile Matthew Matobole [1], Leonardo Joaquim van Zyl [1,*], Shirley Parker-Nance [3,4], Michael T. Davies-Coleman [2] and Marla Trindade [1]

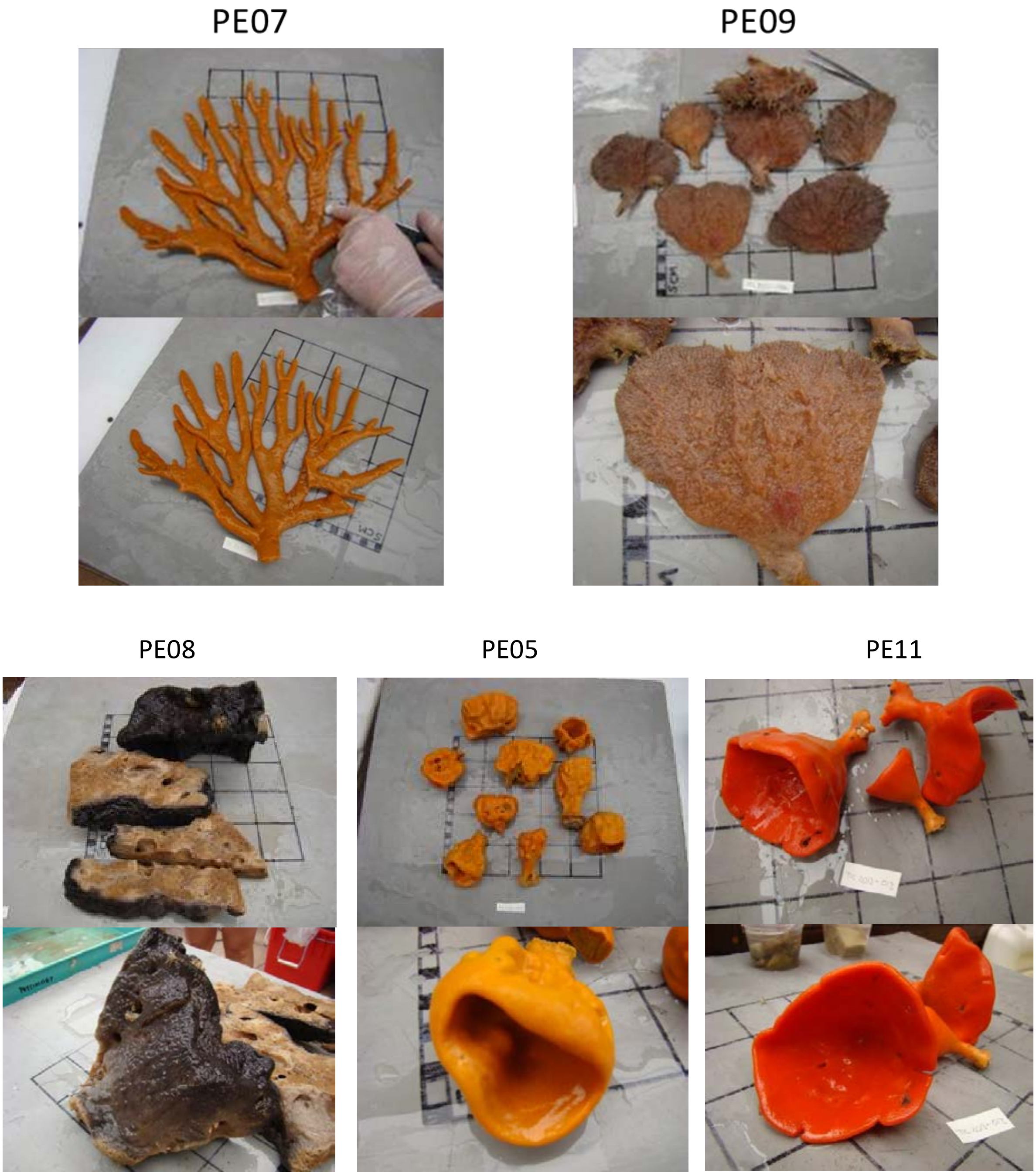

Figure S1. Sponges used in this study: PE05 - *Waltherarndtia caliculatum*, PE07 - *Higginsia bidentifera*, PE08 - *Spongia* sp., PE09 - *Isodictya compressa*, PE11 - *Axinella* sp.

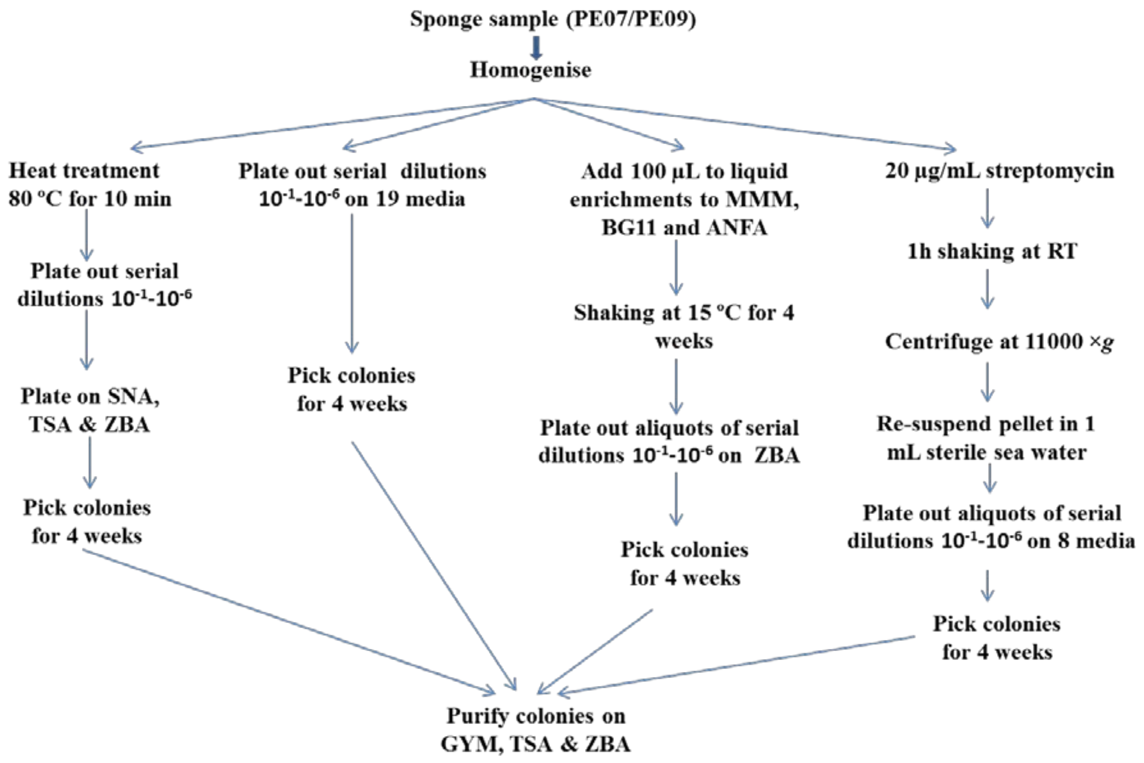

Figure S2. Flow diagram showing the culturing process used in this study.

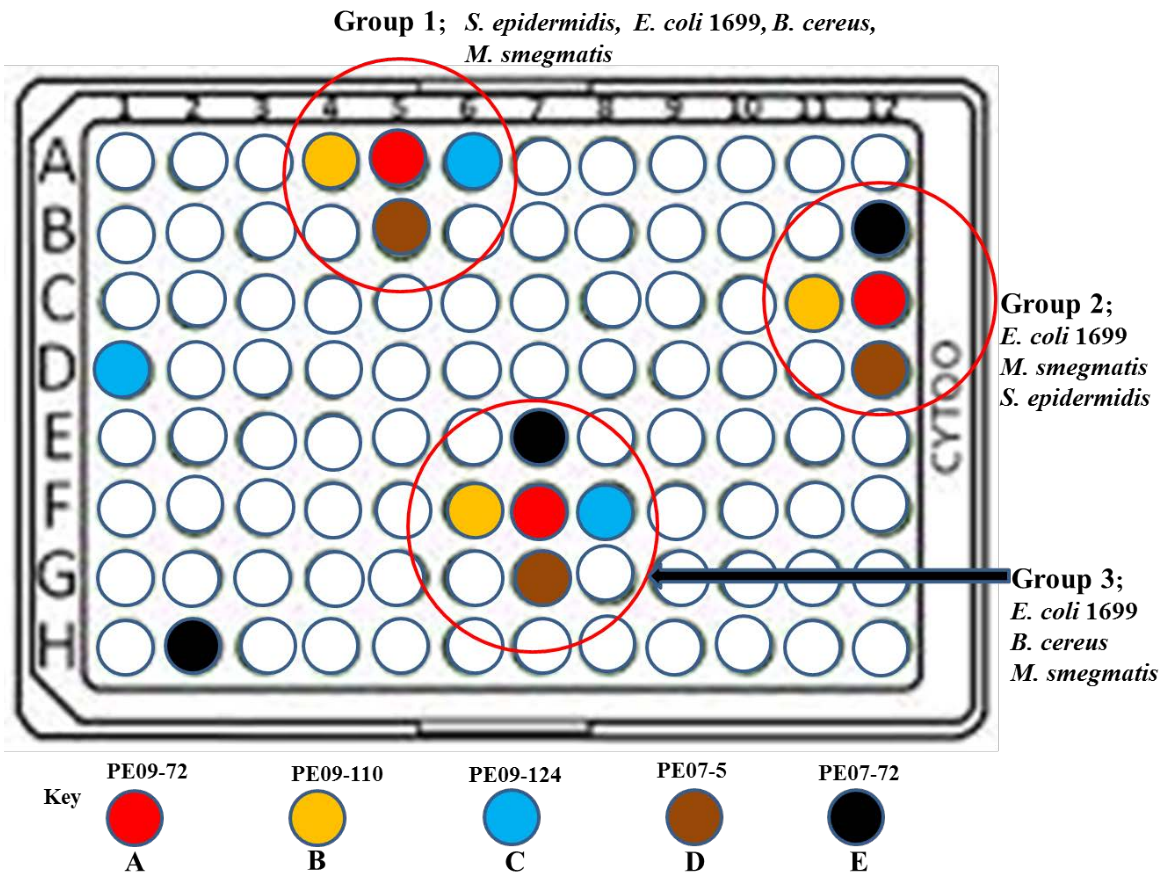

Figure S3. 96-well plate setup for matrix screening. Bioactivities of the isolate PE9-72 when cultured on ($NaNO_3$, 0.5 mM phosphate, mannitol and heat shock) under co-culture conditions with 3 and 4 "immediate" neighbouring isolates in 96 well-plate setup.

Table S2. Panel of 29 isolates selected for the matrix study; 12 isolates which showed antibacterial activities after primary screening and 17 isolates which did not show any bioactivities

Isolate	Identity (%)	Closest hit/relative (as present in EzTaxon)	Phylum/class
		**Activities in prior screening**	
PE07-5	99.7	*Bacillus vietnamesis* B-23890	*Firmicutes*
PE07-106	99.5	*Oceanobacillus picturae* LMG 19492	*Firmicutes*
PE07-133	99.7	*Micrococcus yunnanensis* YIM 65004	*Actinobacteria*
PE07-144	99.7	*Bacillus vietnamensis* B-23890	*Firmicutes*
PE07-172	98.8	*Sporosarcina aquimarina* SW28	*Firmicutes*
PE07-200	99%	*Staphylococcus warneri* ATCC 27836	*Firmicutes*
PE09-110	99.8	*Citricoccus nitrophenolicus* PNP1	*Actinobacteria*
PE09-124	98.9	*Kushneria pakistanensis* NCCP-934	*Gammaproteobacteria*
PE09-140	100	*Pseudomonas fulva* NBRC 16637	*Gammaproteobacteria*
PE09-142	NA	NA	
PE09-221	96.5	*Aeromicrobium erythreum* NRRL B-3381	*Actinobacteria*
PE09-229	99.9	*Halomonas titanicae*	*Gammaproteobacteria*
		**No prior activities**	
PE07-37	99.5	*Bacillus hwajinpoensis* SW-72	*Firmicutes*
PE07-72	99.9	*Brevibacterium frigoritolerans* DSM 8801	*Firmicutes*
PE07-103	99.5	*Bacillus kochii* WCC 4582	*Firmicutes*
PE07-124	99.8	*Brevibacterium frigoritolerans* DSM 8801	*Firmicutes*
PE07-136	99.7	*Lysinibacillus fusiformis* NBRC 15717	*Firmicutes*
PE09-4	99.9	*Staphylococcus saprophyticus* ATCC 15305	*Firmicutes*
PE09-55	100	*Pseudovibrio ascidiaceicola* DSM 16392	*Alphaproteobacteria*
PE09-72	99.9	*Bacillus zhangzhouensis DW5-4*	*Firmicutes*
PE09-77	NA	NA	
PE09-103	100	*Pseudovibrio ascidiaceicola* DSM 16392	*Alphaproteobacteria*
PE09-116	NA	NA	
PE09-163	99.9	*Staphylococcus saprophyticus* ATCC 15305	*Firmicutes*
PE09-180	98.7	*Marinilactibacillus piezotolerans* DSM 16108	*Firmicutes*
PE09-222	100	*Pseudovibrio ascidiaceicola* DSM 1692	*Alphaproteobacteria*
PE09-250	99.9	*Paenibacillus glucanolyticus* DSM 5162	*Firmicutes*
PE09-267	NA	NA	
PE09-274	99.8	*Staphylococcus saprophyticus* ATCC 15305	*Firmicutes*
